# Anticipatory, Chronic, and Imminent: A Typology of Insecurities Underlying Protracted Conflict Displacement and Its Implications

**DOI:** 10.1111/padr.70049

**Published:** 2026-04-04

**Authors:** STEPHANIE M. KONING, GOLEEN SAMARI, ABIGAIL WEITZMAN

**Affiliations:** University of Nevada, Reno, Reno, NV 89557.; University of Southern California, Los Angeles, CA 90007.; University of Texas at Austin, Austin, TX 78712.

## Abstract

Protracted armed conflicts increasingly drive long-term displacement, yet demographic frameworks often treat forced migration from conflict settings as a response to acute, singular events. This study introduces a typology of displacement grounded in the tempo and form of conflict-related insecurities—anticipatory, chronic, and imminent—and examines their consequences for women displaced from Myanmar to Thailand. Using survey data from 390 women, latent class analysis identified distinct pre-displacement insecurity profiles and linked them to post-displacement outcomes, including parallel insecurity profiles, mobility constraints, and labor exploitation. We also examined legal status and residence type as additional understudied yet policy-relevant post-displacement outcomes. Most respondents fled under anticipatory or chronic conditions rather than acute violence, underscoring that displacement from protracted conflict settings is often propelled by cumulative structural harm. Regression models showed that women who fled under chronic or imminent insecurity were substantially more likely to experience continued precarity after migration, including camp residence and insecure legal status. These findings highlight the need for temporal nuance in migration theory and humanitarian policy, recognizing conflict displacement as a prolonged process with enduring insecurities for women, families, and future generations, rather than a discrete event.

## Introduction

Armed conflict today is increasingly protracted, producing displacement that is long-term, cyclical, and structurally embedded (Zetter 2011). Most of the world’s conflict-displaced populations live in long-term exile, without permanent legal status, or a home. By the end of 2023, more than 117 million people were forcibly displaced worldwide, including 27.2 million refugees and 43.4 million people in refugee-like situations—a 7% increase from the previous year (UNHCR 2024). Most refugees reside in low- and middle-income countries neighboring their own, and two-thirds live in protracted situations (UNHCR 2024). An additional 1.3 million displaced people are stateless, lacking citizenship and the protections it affords (UNHCR 2024). Despite the rising global scale of forced migration, demographic research has only recently begun to grapple with the population-level consequences of protracted and episodic forms of forced migration (Massey 2023). Existing migration theories often frame displacement as a response to singular, acute events—such as sudden violence, political rupture, or economic collapse (Lee, Carling, and Orrenius 2014). However, such frameworks rarely account for the temporal accumulation or layering of insecurities that unfold over months or years in protracted conflict settings.

The concept of structural violence provides a valuable lens for understanding these dynamics (Farmer et al. 2004; Galtung 1996). Galtung (1969, 168) famously defined structural violence as “present when human beings are being influenced so that their actual somatic and mental realizations are below their potential realizations.” In other words, structural violence occurs when social structures or institutions systematically limit individuals or groups from achieving their full potential, leading to avoidable harm, disadvantage, or suffering that may even surpass recognition at the individual level. As such, structural violence remains distinct from the specific mass violence resulting from collective action that is generally ascribed to armed conflict, even as it also acts as a cause and consequence of armed conflict (Brück, Justino, and Verwimp 2013). Protracted conflicts rarely involve uninterrupted mass violence. Instead, they fuel structural violence that, in turn, underlies long periods of simmering insecurity, episodes of heightened repression, and eventual escalation to acute harm. These threats emerge not only from armed actors but also through infrastructure collapse, legal precarity, and socially embedded forms of exclusion (Dubal, Samra, and Janeway 2021; Weitzman and Huss 2024). Like the slow-onset stressors of climate change, such threats unfold unevenly over time—shaping both the timing and trajectory of displacement (McMichael, Schwerdtle, and Ayeb-Karlsson 2023).

Thailand offers a case in point. Decades of armed conflict in Myanmar’s Shan State—driven by clashes between the Tatmadaw (Myanmar’s military), ethnic armed organizations, and competing militias—have produced cycles of violence, militarization, and forced recruitment along-side entrenched poverty and land dispossession (Lintner 1984; Silverstein 1958; Smith 2007). These dynamics have been compounded by overlapping political instability, illicit economies, and the absence of durable ceasefires in many areas, sustaining both acute episodes of fighting and chronic livelihood insecurity (Meehan 2015; South 2008). Northern Thailand has long served as a destination for those fleeing these conditions, offering relative safety but rarely durable protection; most cross-border migrants remain without formal refugee status, facing irregular legal standing, precarious employment, and ongoing risk of arrest or deportation (Banki and Lang 2016; Grundy-Warr and Wong Siew Yin 2002; Koning 2019).

Consistent with structural violence theory, this study offers a novel typology of displacement-inducing insecurities in protracted conflict contexts. Drawing on data from displaced populations along the Thailand–Myanmar border and applying an inductive latent class analysis (LCA), we conceptualize premigration insecurities as anticipatory, chronic, or imminent. These categories reflect variation in both intensity and the timing of risk under which mobility decisions are made. They differentiate between anticipatory threats and insecurity, ongoing structural harm, and urgent life-threatening violence. Using LCA, we show how these temporally distinct profiles shape both the initiation of displacement and subsequent patterns of vulnerability post-displacement. To assess such patterns, we elucidate a parallel typology of post-displacement insecurities, along with two salient factors underrepresented in forced migration scholarship, legal status and residence type, to consider individuals who have not achieved refugee status and those living outside a camp.

## Background

### Rethinking push factors in forced migration

One of the most fundamental theories in migration is the “push–pull” factor theory, which posits that migration is a macro-level function of factors like income and opportunity gaps between origin and destination countries. Various economic, environmental, and demographic factors are assumed to push people out of places of origin and pull them into destinations (De Haas, Castles, and Miller 2019; Massey et al. 1993). It is commonly argued that the “push–pull” factors theory is primarily applicable to voluntary migrants and not as well-suited for forced or conflict-driven displacement (Klaus and Pachocka 2019). Yet, scholars have extended the “push–pull” factor model to conflict and forced displacement. Kunz (1973) expanded push factors to a typology accounting for how people are pushed and coerced to leave their country of origin to save their own lives. He distinguished between acute refugee movements following sudden events and anticipatory refugee movements when more planning is involved before escape. Others have further extended the “push–pull” factor model to conflict and forced displacement, presenting theories like the “push out–push back” approach which focus on push factors from migrants’ country of origin, initial destinations, and countries of the Global North (Klaus and Pachocka 2019). Primary push factors driving forced migration in the 21st century are conflicts, development policies and projects, and natural disasters, often in combination—and these can also intersect with underlying economic push–pull dynamics (Matsui and Raymer 2020; Mitchell and Pizzi 2021; Raleigh 2011). None of these frameworks, however, explicitly account for how the temporal nature of conflict and forced displacement shapes push factors.

Conventional frameworks in forced migration often conceptualize conflict-related violence as a binary or acute phenomenon—conflict either “breaks out” or it does not (Lee, Carling, and Orrenius 2014). Within these paradigms, push factors are treated as static and event-driven, triggered by sudden political upheavals, massacres, or economic collapse. Yet displacement frequently results not from a single episode, but from the cumulative layering of insecurities that intensify over time (Cantor 2024; Farrell and Schmitt 2012; Schmeidl 1997). For example, out-migration from Venezuela began increasing as public safety and social conditions eroded, several years *before* the most severe inflationary crisis (Weitzman and Huss 2024). Similarly, qualitative studies of Syrian displacement highlight that out-migration from conflict zones shifted over time with evolving conditions inside Syria—such as warfare intensity, roadblocks, and economic insecurity—as well as border closings and immigration policies in receiving countries (Arar and FitzGerald 2022, 92–103).

Conflict and conflict-induced displacement produce both acute and protracted uncertainty (Horst and Grabska 2015). The protractedness of many conflicts and, by extension, of displacement creates liminal situations for refugees and internally displaced persons (IDPs), in which migration decisions and waiting play central roles. The type of precariousness that conflict and displacement create reconfigures society, often push-pulling people affected in contradictory directions (Klaus and Pachocka 2019). In these complex settings, events occur at varying speeds and in unpredictable patterns, thus, violence rarely manifests uniformly nor instantaneously (Cantor 2024). Instead, conflict violence exhibits considerable heterogeneity in intensity, form, duration, and visibility, ranging from subtle yet pervasive forms of discrimination, economic deprivation, and legal exclusion, to overt acts of aggression by state and non-state actors (UNHCR 2024). Understanding this spectrum of violence is critical, as it shapes both the occurrence and timing of displacement and other related decision-making processes (Brück, Justino, and Verwimp 2013).

The prevalent binary categorizations, such as “forced vs voluntary” or “economic vs political,” prove inadequate when applied to these nuanced realities (Zetter 1991). Such typologies overlook the cumulative temporal evolution of threats to security that characterize many protracted crises. They also overlook the fact that migration occurs along a “continuum of volition” (Fussell 2012). The existence of a continuum implies that anticipatory migration to avoid conflict may simultaneously be forced and voluntary, for example, to get out of harm’s way rather than risk harm before migrating. Further, binary categorizations are heavily reliant on borders as a defining reality of displacement when in protracted crises, borders are in fact far more permeable than these dichotomies imply. The legal distinction between the different categories of forced migrants, which is border reliant, is largely artificial. In contrast, structural violence theory (Farmer et al. 2004; Galtung 1969, 1996) provides a more dynamic framework, highlighting how systemic conditions like chronic insecurity, legal marginalization, and social exclusion operate below the threshold of immediate crisis. This theoretical approach foregrounds how prolonged structural inequities and chronic violence systematically erode individuals’ safety, dignity, and livelihoods, ultimately compelling migration even in the absence of a discrete violent event. Taken together, these insights underscore that structural violence is not only a condition that sustains displacement but also a driver of conflict, generating a self-reinforcing cycle of insecurity whose evolving tempo demands closer attention across protracted displacement processes.

### Temporal dimensions of insecurity in protracted conflict displacement

Building on the theory of structural violence, we argue that temporality significantly shapes displacement dynamics. While we often think of conflict induced displacement as a singular and, perhaps, extended event, the reality is one of much more temporal complexity (Etzold and Fechter 2022). Individuals within the same conflict setting may experience insecurities and decide to migrate according to highly varied timelines, thresholds of risk perception, and anticipatory calculations. While some migrate preemptively in response to early indicators or warnings of escalating violence, others endure prolonged periods of structural oppression and insecurity before displacement becomes inevitable. Still, others only flee in response to immediate existential threats and when immediate harm becomes unavoidable.

Protracted conflicts inherently create conditions in which chronic risks coexist alongside acute episodes of violence, intensifying progressively and unevenly over time. Threats to human security are not experienced uniformly, nor are they experienced all at once or homogenously distributed. Instead, insecurity unfolds episodically and cumulatively, shaped by political repression, systematic discrimination, livelihood degradation, legal precarity, and shifting patterns of militarization. As Massey (2015) points to in the case of the United States and Mexico, the actions of “politicians and self-serving bureaucrats” ultimately shifted circular migration patterns to an acceleration of undocumented population growth. Systemic threats, including those of political origins like the “Latino Threat Narrative,” shape migratory flows in meaningful ways. Vigh (2008) used the term “chronicity” to analyze the persistence and normalcy of violent crises in the lived experiences of affected populations. While this notion elevates the temporal dimensions of violence, this perspective seemingly implies that displaced people have accepted the conditions of conflict and crises. Recognizing the temporal complexity of these threats in conjunction with migration outcomes is crucial for understanding the differential vulnerability, decision-making, and migration patterns within conflict-affected populations.

Our proposed typology explicitly integrates the dimension of temporality to capture variations in the nature and timing of displacement-inducing insecurities. We distinguish between anticipatory, chronic, and imminent displacement pathways to empirically model how timing and form of threats to human security shape migration decisions in such settings. By grounding our analysis in structural violence theory, we emphasize that displacement frequently results not from explosive or exceptional violence, but rather from prolonged, cumulative, and structurally embedded conditions (Farmer et al. 2004; Galtung 1996). In doing so, we position timing as central, rather than incidental, to understanding displacement dynamics, subsequent vulnerabilities, and why certain groups remain vulnerable long after crossing a border.

### A typology of displacement-driving insecurities: The empirical case of protracted displacement from Myanmar to Thailand

Forced migration from protracted conflict settings is frequently simplified around singular violent events or clearly identifiable crises. Such oversimplifications obscure how insecurities accumulate, escalate, and intersect over time, prompting diverse displacement trajectories. Drawing on prior research that examined structural violence across displacement trajectories along the Thailand–Myanmar border (Koning 2019), this study introduces a typology of displacement-driving insecurities that foregrounds their temporality, escalation, and immediacy at the point of departure.

While previous research showed how structural violence shapes long-term outcomes and becomes embodied over the life course, including post-migration hardships, this study uniquely prioritizes the conditions of forced migration, specifically temporal patterns of violence and insecurity surrounding migration. Using survey data from displaced women near the Thailand–Myanmar border, we apply LCA to empirically identify three temporal profiles of conflict-related insecurities and assess their predictive power for post-displacement insecurity, including arrest risk, housing conditions, and legal status.

We delineate the following typology of displacement-driving insecurities (Figure 1), expanding on the kinetic model theorized (but not empirically examined) by Kunz (1973) and drawing from prior interview and survey research that assessed clustering of violent or otherwise stressful life events reported by participants (Koning 2018):

1. *Anticipatory insecurity displacement*: Migration prompted by growing insecurity and perceived escalation of violence, akin to what Kunz (1973) calls anticipatory refugee movements. Individuals flee before experiencing direct harm, often responding to early warning signals. For example, some respondents fled after a relative was forced into military labor—a sign of deteriorating safety rather than immediate victimization.
2. *Chronic insecurity displacement*: Migration after years of structural oppression and low-level, persistent insecurity. This has been underrepresented in prior migration theories. These individuals often experience long-term military occupation, property loss, or social exclusion. For example, some study participants described gradual dispossession over several years before displacement became unavoidable.
3. *Imminent insecurity displacement*: Abrupt migration triggered by acute, life-threatening violence such as home destruction, direct attack, or forced evacuation, similar to what Kunz (1973) calls acute refugee movements. One illustrative case is a woman fleeing after a military raid destroyed her village, leaving no safe options for return.

This typology advances demographic and migration research by explicitly incorporating temporal variation in insecurities experienced in protracted conflicts as a core explanatory dimension and push factor. By moving beyond binary frameworks, our study elucidates how protracted conflicts produce diverse, temporally structured patterns of oppression and insecurity, informing both displacement timing and subsequent vulnerability. Moreover, the chronic dimension of protracted conflict and insecurities is brought to the forefront of the discussion of “push” factors in forced migration. We examine how pre-displacement insecurity profiles unearthed by LCA and distinguished by their timing, duration, and intensity, shape post-migration insecurity, legal precarity, and living conditions. This dual focus on classification and consequence offers a novel contribution to conflict and displacement studies by linking when and how insecurities accumulate to the enduring risks migrants face after crossing borders.

## Data and methods

### Study setting and population

This study draws on cross-sectional survey data collected between 2016 and 2017 in two districts along Thailand’s northern border with Myanmar, in Chiang Mai Province (Koning 2018). The study sites are long-standing destinations for migrants from conflict-affected areas of Shan State, Myanmar. The first district, historically linked to Shan State through an open border until 2002, retains dense transborder kinship and ethnic ties and hosts the only informal refugee camp along this segment of the border. The second district is a larger agricultural and commercial hub, where migrant labor—documented and undocumented—supports key sectors including agriculture, construction, and manufacturing. In both areas, migrants from conflict zones frequently register as economic migrants or remain unregistered to preserve livelihood opportunities, underscoring the interplay between displacement and labor migration. These characteristics make the sites analytically relevant for examining the layered insecurities, legal precarity, and livelihood dynamics that typify protracted displacement in borderland settings broadly.

Within each study site district, one sub-district per site was selected for inclusion. Sub-districts were selected to reflect variation in displacement timing, conflict exposure, and residential setting. The study population includes women aged 18 and older who had experienced pregnancy in the previous five years and resided in rural villages, informal camps, or worksites. Among women in the sample who were born in Myanmar, mean age at displacement was 16.5, and most exposures to conflict-related violent events occurred between ages 0 and 21 (Koning et al. 2025).

Because no sampling frame existed for all eligible participants, the research team conducted household canvassing across all accessible communities within the selected district boundaries. This process was supported by partnerships with subdistrict health staff, village leaders, and community-based organizations, who provided complete lists of registered households and worksites. Canvassing further enabled the inclusion of participants from unregistered households and worksites. The final sample comprised respondents from 76 community clusters, spanning 37 government-registered villages, adjacent worksites, and one camp. At recruitment, women completed a brief enrollment survey and anthropometric measures, followed by scheduled at-home interviews. Participation was high (99%) among the 824 eligible women recruited (Koning et al. 2021). Among women recruited for study participation, 577 completed at-home interviews, including questions used in the current study (70.0%). Women born in Thailand were excluded from the current study analysis, leaving 390 migrant women who fled Myanmar in their lifetime in the current study sample.

All interviews were conducted in private by trained interviewers in local languages. Informed consent was obtained from all participants. Ethical approval was provided by the Chiang Mai Provincial Ministry of Public Health and the University of Wisconsin–Madison IRB. Participants were compensated for their time with household gifts and were offered referrals to no-cost mental health and social services following interview completion.

### Survey design and measurement

The survey instrument was informed by 2 years of preparatory ethnographic work and cognitive interviews (Koning 2018). It included several modules on violence exposure, migration history, life-course adversity, reproductive health, and resettlement conditions.

#### Pre-displacement insecurities.

We used 24 binary indicators, drawing from an original 25 questions, derived from a life event inventory tailored to the Myanmar conflict context (listed in Supplement Table A1; one life event was dropped due to zero reports). Items included were based on pre-survey field work and qualitative interviews about conflict-related life experiences in Shan State. These captured whether respondents had ever experienced specific conflict- or displacement-related events, including experiences of military occupation and control (e.g., invasion or conscription), being forced from one’s home (e.g., having to flee or nearby explosions), and deprivation (e.g., no food or clean water). These indicators were used as inputs in the first LCA, aimed at identifying insecurity typologies at the time of migration. Internal consistency for the full inventory was high (Cronbach’s *α* = 0.846).

#### Post-displacement insecurity.

Post-displacement insecurities and stressors were captured using 14 out of 15 binary indicators from a separate Thailand-based life event inventory survey instrument (one item was dropped due to zero reports). These items were also based on pre-survey field work and qualitative interviews asking about threats, insecurities, and stressors in the study context (listed in Supplement Table A1). Experiences included labor exploitation, fear or mistreatment by brokers or authorities, hiding from arrest, and deprivation of shelter or care. Items from this inventory was used to identify latent profiles of post-displacement vulnerability via a second LCA. Internal consistency for this set of items was high (*α* = 0.754).

#### Migration and resettlement characteristics.

Key contextual variables were used to describe migrant characteristics and circumstances. Legal status was measured based on documentation held and categorized as holding either legal permanent resident status (i.e., a legal “alien” resident allowed to live and work in Thailand, with possible permission to apply for citizenship in the future), legal migrant documentation (i.e., a visa and/or work permit allowing one to live and/or work in Thailand, respectively), or temporary status (e.g., a 10-year permission card to live—but not work—in Thailand as a temporary asylum seeker) or no documentation. These legal statuses are listed in order of increasing precarity, generally. Housing type was coded as private home, worksite, or (informal) camp—also following an order of increasing precarity.

#### Socio-demographic characteristics.

Educational attainment was measured based on highest completed level of schooling reported. This was categorized based on general levels of schooling aligning with years, with primary school including grades 1–6 and secondary school including higher grades, which is consistent in Thailand- and Myanmar-based schools. Based on the sample distribution of schooling completion in the study population and related socioeconomic advantage, this variable was categorized as completing less than all primary school (i.e., less than grade 6), all primary school (i.e., completing grade 6), and any secondary school or higher (i.e., completing at least grade 7). Binary indicators of income source were coded from both data collection stages: open-ended responses at enrollment (anthropometric measurement stage) and pre-coded categories at the at-home interview. Responses were harmonized to indicate a single primary work area (e.g., agriculture, manufacturing, construction), with volunteer roles (e.g., village health volunteer) excluded so that categories remained mutually exclusive. Cultural identity was also reported, specifically what cultural and ethnic group the respondent identified with most strongly, categorized as Thai, Shan, or other highland minority group. Respondent age and age at time of displacement were also recorded, from which duration of years in Thailand was calculated for sensitivity analyses.

### Analytic strategy

We conducted LCA, a person-centered, model-based clustering technique, to empirically identify unobserved subgroups of displaced women based on patterns of insecurity exposure before and after migration. Our approach inductively uncovers subgroups based on multiple observed characteristics at once, and in this way, helps to reveal meaningful clustering of pre-migration insecurities as well as the clustering of post-migration insecurities (separately). All models were estimated using the gsem command in Stata 18, specifying a Bernoulli family with a logit link for binary indicators. To account for potential nonindependence and improve model stability, we used robust standard errors (vce(robust)) and imposed a nonzero tolerance threshold (nontolerance). Each model incorporated full information maximum likelihood (FIML), which allowed for the inclusion of respondents with partial missing data on indicator variables. Among the 390 participants in the current study analysis, item response ranged from 77% to 100% across variables, with all respondents contributing at least one life event measure (see Supplement Table A2).

Model selection was guided by empirical fit and interpretability; our substantive interpretation of the resultant classes was guided by existing theory and literature. The first LCA, using 24 pre-displacement indicators, identified a three-class solution representing distinct insecurity-based displacement typologies: Anticipatory Insecurity, Chronic Insecurity, and Imminent Insecurity Displacement. The second LCA, using 14 post-displacement indicators, produced a three-class model reflecting different patterns of ongoing insecurity in Thailand: Stable/Integrated Migrants, Vulnerable Migrants, and Mobility-Threatened Migrants. Model fit was assessed using the Akaike information criterion (AIC). We also examined class size, average posterior probabilities, and cross-class separation. Entropy values were not available via gsem, but predicted class probabilities and confidence intervals were used to evaluate class distinctiveness. Participants were assigned to classes based on the highest posterior probability using predict with classposteriorpr. Class profiles were summarized using estimated class-specific marginal means.

Cross-tabulations and descriptive statistics were used to examine how displacement typologies and post-displacement profiles aligned with migration histories and resettlement characteristics. To further assess the relationship between pre-displacement insecurity typologies and post-displacement outcomes, we estimated a series of multinomial logistic regression models. These models examined the predictive value of pre-displacement latent class membership on three key post-migration outcomes: post-displacement insecurity profile, legal status, and residential setting. All models controlled for respondent education level using a three-category indicator (<primary, primary, secondary+). Models were estimated in Stata 18 using the mlogit command with robust standard errors, and all outcomes were specified using their most secure category as the reference (i.e., Stable/Integrated Migrants, Permanent Legal Status, or Private Residence). For interpretability, we report both relative risk ratios (RRRs) with 95% confidence intervals and post-estimation adjusted marginal probabilities by pre-displacement class membership using the margins command.

Sensitivity checks were conducted to assess robustness. For the LCA, post hoc power calculations suggested our sample (*n* = 390) was adequate for a three-class analysis (Cohen 2013; Westland 2010), and diagnostics supported class stability. For multinomial regressions, power was limited by small subgroup sizes, so we prioritized a parsimonious adjustment strategy (education only) and ran sensitivity analyses with both unadjusted models and models additionally adjusted for duration of displacement.

## Results

### Sample characteristics

The analytic sample included 390 women who had migrated from Myanmar to Thailand during the Myanmar conflict (Table 1). The median age of participants was 29 years (Q1 = 24, Q3 = 34). A majority identified as Shan (75%), while 20% identified as other highland ethnic minority groups, and a small minority (5%) as Thai. Most participants had low levels of formal education: nearly three-quarters (73%) had not completed primary school. Participants’ legal status varied, with 37% having permanent residency, 39% holding temporary or migrant permits, and 24% lacking any formal legal documentation. Housing conditions also differed, with 4% residing in informal camps, 68% in rural private homes, and 28% in worksite housing. Reported income sources were diverse, with agricultural wage labor (36%) and factory work (20%) most common. Supplement Table A2 includes additional sample characteristics.

### Typologies of displacement-related insecurity

LCA of 24 binary conflict exposure indicators identified three distinct profiles of pre-displacement insecurity, summarized in Figure 2 and Supplement Table A3. The selected three-class solution showed good fit (AIC = 4067) relative to the alternative two-class model (AIC = 4259). The final model also demonstrated high classification certainty (average posterior probabilities: Class 1 = 0.96; Class 2 = 0.94; Class 3 = 0.94) and strong class separation (normalized entropy = 0.90).

The largest class (61%) members, based on those with a higher predicted probability of being in this class than any other class, experienced minimal risk of direct violence prior to migration. This class was labeled Anticipatory Insecurity Displacement. This group was characterized by low probabilities of reported violence or deprivation, with anticipatory displacement likely driven by social or environmental signals of escalating risk. A second class (31%) showed elevated risk exposure to sustained threats such as military occupation, property seizure, or livelihood degradation, and was labeled Chronic Insecurity Displacement. These respondents demonstrated risk of indirect and structural violence across multiple domains, consistent with long-term exposure to insecurity. The smallest group (8%) reflected acute displacement conditions, including discrete events such as direct attack, forced conscription, and home destruction. This class, labeled Imminent Insecurity Displacement, encompassed multiple forms of victimization and fleeing specific events or crisis-like conditions.

### Post-displacement insecurity profiles

LCA of 14 binary post-displacement insecurity indicators identified three distinct experiential profiles, summarized in Figure 3 and Supplement Table A4, with improved fit (AIC = 1771) relative to the alternative two-class model (AIC = 1859). Model diagnostics supported this specification, with high normalized entropy (mean = 0.96) and strong posterior probabilities across all classes (Class 1 = 0.99, Class 2 = 0.95, Class 3 = 0.97), indicating strong class separation and assignment precision.

The largest class, Stable/Integrated Migrants (75%), exhibited low predicted probabilities across nearly all indicators of post-displacement insecurity. Members of this class exhibited limited risk exposure to labor exploitation (e.g., 6% unfair pay; 0% documentation issues), travel-related fear (≤1%), and material deprivation (≤2%). These patterns suggest relative integration and security compared to the other groups. The second and smallest class, Vulnerable Migrants (5%), exhibited elevated exposure risk to labor-related exploitation (e.g., 73% unfair pay; 34% documentation issues; 30% fear of brokers) and unmet basic needs (26% no care; 20% lacked food or drinking water). They also experienced the highest predicted probability of direct or indirect arrest (39% for self and 21% for family), yet their probability of fear, hiding, or not traveling to avoid authorities was not the highest. The third class, Mobility-Threatened Migrants (20%), faced high structural and situational insecurities, including the highest probabilities of travel-related fear (97%) and avoidance (93%), and hiding to evade arrest (90%). Probabilities of labor exploitation and unmet needs were lower in this class than the Vulnerable Migrant class, which may be due to overall higher constraints on mobility and work.

### Continuity of precarity: Linking pre- and post-displacement insecurities

Cross-tabulations of pre- and post-displacement latent classes (Supplement Table A5) revealed significant alignment between pre- and post-displacement insecurity (*χ*^2^(4) = 23.9, *p* < 0.001; Fisher’s exact *p* < 0.001). Among those in the Anticipatory Insecurity Displacement class, 81% were classified as Stable/Integrated post-migration, 4% as Vulnerable, and 15% as Mobility-Threatened. Chronic Insecurity Displacement was associated with more varied trajectories: 66% Stable/Integrated, 5% Vulnerable, and 29% Mobility-Threatened. Imminent Insecurity Displacement was linked to the highest proportion experiencing post-migration precarity—38% (19% Vulnerable, 19% Mobility-Threatened). These patterns, shown in Figure 4, highlight both continuity and divergence in precarity across displacement processes. Distributions of education, age at interview, and age at displacement did not significantly differ across insecurity classes, though there was modest evidence of age variation by pre-displacement class (Supplement Table A5).

Adjusted multinomial logistic regression models revealed that pre-displacement insecurity typologies were significantly associated with multiple post-displacement outcomes, even after accounting for education level (Table 2). Compared to women classified with Anticipatory Insecurity Displacement, those with Chronic Insecurity were over twice as likely to be classified as Mobility-Threatened migrants (RRR = 2.61, 95% CI: 1.52–4.48), and those with Imminent Insecurity Displacement had over six times the risk of assignment to the Vulnerable class (RRR = 6.70, 95% CI: 2.11–21.24). While no significant differences by threat class were observed for legal status or worksite residence, individuals classified in the Imminent Insecurity Displacement were nearly eight times more likely to reside in camp settings post-migration than private housing (RRR = 7.76, 95% CI: 2.26–26.63).

These adjusted patterns are further summarized as predicted probabilities estimated based on each multinomial regression model using marginal standardization (Supplement Table A6). Anticipatory Insecurity Displacement was associated with the highest probability of Stable/Integrated post-displacement status (81%), while Imminent Insecurity was associated with elevated risks of both Vulnerable (20%) and Mobility-Threatened (18%) classification. Compared to other groups, the Imminent group had the highest probability of residing in a camp (16%; 95% CI: 4%–28%) and the lowest probability of living in a private home (56%; 95% CI: 38%–74%). Although statistically significant differences were not detected in legal status outcomes, the Imminent group also had the highest estimated probability of no documentation or temporary status (32%; 95% CI: 16%–48%). Figure 5 visualizes these adjusted probabilities across pre-displacement insecurity classes and outcomes jointly.

The sensitivity checks that reran the multinomial regressions of post-displacement outcomes on pre-displacement insecurity class without adjusting for education resulted in all coefficients consistent in magnitude and statistical significance (Supplement Table A7). The second set of sensitivity analyses that adjusted for both education and duration of displacement resulted in consistent coefficient magnitudes and statistical significance for all coefficients that were statistically significant in the main analyses adjusting for education only (Supplement Table A8).

## Discussion

This study introduces a novel typology of displacement based on the tempo and form of insecurity, distinguishing anticipatory, chronic, and imminent pathways of forced migration in protracted conflict. Using original survey data from women displaced along the Thailand–Myanmar border, we applied LCA to identify subgroups defined by pre- and post-migration insecurities. These dual typologies show that the timing and form of insecurity—beyond its intensity—critically structure the displacement process and its consequences. Importantly, the findings illustrate a patterned continuity of insecurity across borders: while some migrants escape direct violence, others experience its continuation, in new forms, after crossing an international border.

Whereas traditional “push–pull” frameworks often conceptualize migration as event driven, our data indicate that most respondents migrate under latent or chronic insecurities. Our findings align with work that poses a socioecological framing of “push–pull” factors at the micro, meso, and macro levels with circular and nonlinear patterns of migration (Qi and Bircan 2023). Specifically, more than half the study participants left amidst early violence escalation, responding preemptively rather than reactively, while another third fled after enduring prolonged periods of oppression and instability. This also underscores the necessity of expanding forced migration theory beyond binary, event-centric frameworks to include accumulated structural violence, chronic oppression and insecurity, and temporally layered risks as key push factors. In particular, the results affirm structural violence theory, emphasizing that conflict displacement arises not only from explicit acts of acute violence but equally from slow and cumulative degradation of safety, dignity, and livelihoods.

Study results align with behavioral models of migration that emphasize how migration journeys unfold in complex, iterative ways, shaped by entangled and sequential decision-making—from forming an initial intention, to making a decision, and ultimately undertaking the move (Klabunde et al. 2017; Kley 2017). They also raise important questions for future research on who is more likely to leave when—whether some individuals depart earlier in anticipation of escalating conflict, only after prolonged insecurity, or primarily in response to imminent threats. For instance, while the anticipatory insecurity group reported relatively few direct violent life events, they nonetheless fled a region experiencing a protracted armed conflict spanning over half a century, suggesting that departure decisions can reflect broader contextual threat environments as much as individual experiences. It is also possible that individuals able to leave earlier had greater socioeconomic resources, family networks, or other unobserved capacities (e.g., resilience or risk tolerance) that facilitated mobility before acute threats escalated, which may partly explain their relative post-migration stability. Moreover, although our adjusted models account for education as one dimension of social position, broader questions remain about how pre-displacement social positioning shapes both the type of insecurities individuals face and their trajectories after displacement. Future work that more explicitly examines the intersection of social position, temporality of migration, and cumulative insecurity will be crucial for clarifying the mechanisms behind these heterogeneous migration pathways. We did not find strong differences in age or timing of displacement across insecurity classes. However, because our study sampled women with specific age-related eligibility, generalizability is limited, and future research should examine whether life stage at migration conditions displacement trajectories and post-migration outcomes.

Our findings contribute to the small but growing body of work challenging conventional forced migration paradigms by showing that immediate harm drives only a minority of migration decisions in protracted conflicts. For instance, studies of Venezuela find that out-migration increased gradually for several years before peaking in 2018–2019, suggesting anticipatory movement (Weitzman and Huss 2024). In the Myanmar case, a substantial portion of respondents also out-migrated anticipatorily, reporting less direct exposure to harm than others—likely owing to their migration timing. By contrast, one-third of our sample experienced chronic insecurity before migrating, including oppression, loss of housing, and food scarcity. As with cases in El Salvador, Venezuela, and Syria (Arar and FitzGerald 2022; Menjívar 1993; Weitzman and Huss 2024), our results underscore that displacement in protracted conflicts is shaped not only by acute violence but also by degrading economic and social conditions accompanying political turmoil and persecution.

Critically, we also find that migration frequently does not resolve insecurity. That is, precarity rarely ends with migration. Individuals who fled under chronic or imminent insecurity were significantly more likely to face post-displacement hardship, including labor exploitation, arrest risk, and housing instability. Conversely, individuals who migrated proactively, at the early stages of insecurity escalation, demonstrated comparatively better post-migration stability. This distinction underscores the need for attention to the timing of migration outflows from protracted conflicts and humanitarian crises, rather than treating migration alone as a protective act that concludes exposure to harm (O’Malley 2018). Demographic analyses should further recognize migration as a phase within a broader continuum of precarity, rather than as an endpoint to a particular vulnerability.

In addition to the timing and form of pre-displacement insecurity, our findings point to the importance of structural and social factors that shape vulnerability after migration. Residence type is a sensitive indicator of post-displacement precarity: individuals classified with Imminent Insecurity Displacement were significantly more likely to reside in camp settings–which offer temporary protection but confer long-term precarity in protracted situations–and were less likely to live in private homes, even after adjusting for education. These housing patterns suggest persistent instability driven by factors beyond mere legal status, with implications not only for women themselves but also for the families and children they are raising in protracted displacement contexts. As durable solutions are advanced to provide for refugees (Maple and Hovil 2025) and research strives to better understand conflict and forced migration (FitzGerald and Arar 2018), these findings underscore the interconnectedness of pre-migration conditions and post-migration social and structural determinants of long-term displacement vulnerability.

From a policy perspective, these findings have implications for how we assess protection needs and design humanitarian responses in cross-border displacement settings. Individuals who migrate under anticipatory or chronic conditions may not meet conventional asylum criteria yet carry substantial risk burdens, including vulnerability to human rights violations (UNHCR 2024). For example, women who left Myanmar amidst chronic structural oppression were significantly more likely to experience constrained mobility in Thailand, even if they were not directly targeted by acute violence. As such, social integration likely varies by pre-migration threat profile as well. These patterns suggest the need for nuanced protection frameworks that account for the cumulative and structural nature of insecurity rather than relying solely on conventional event-based violence assessments.

This study has several limitations. First, the cross-sectional design limits causal inference about how exposure to insecurity unfolds over time. We were also unable to model precise timing of exposures, and although missingness was generally low, timing-related measures such as age at displacement had higher rates of incomplete response. FIML was used to retain all respondents, but future studies with larger samples and more complete timing data could strengthen these estimates. Second, underreporting of sensitive experiences, particularly gender-based violence, may lead to conservative estimates despite strong rapport with participants. Third, statistical power was constrained by small subgroup sizes, which limited the extent of model adjustment; however, sensitivity analyses suggested results were robust to alternative specifications. Fourth, although canvassing strengthened representativeness by including both registered and unregistered households, some inaccessible households may still have been missed. Finally, while the Thailand–Myanmar border provides a critical setting for studying protracted conflict and irregular migration, findings may not be generalizable to other geographies with different legal regimes, conflict dynamics, or integration frameworks, nor beyond the specific population studied—women aged 18 and older who had experienced pregnancy in the previous 5 years.

Despite these limitations, this analysis expands the literature on “push–pull” factors in conflict-induced forced migration and protracted conflicts by explicitly differentiating displacement according to the temporal accumulation and form of pre-migration insecurities. Traditional push–pull models often inadequately capture the experience of forced migrants, dynamics of protracted conflicts, and ongoing vulnerabilities after migration. This study also demonstrates that the nature of those threats, whether anticipatory, chronic, or imminent, shapes distinct patterns of post-migration precarity. In doing so, the study underscores the need for greater temporal nuance in both demographic research and policy frameworks addressing conflict-driven migration. Displacement is a complex, ongoing process rather than a singular event, elevating the need for research, policy, and humanitarian approaches that address not just the immediate act of migration, but the sustained vulnerabilities faced by displaced populations long after crossing borders. For women in particular, these vulnerabilities reverberate through families and across generations, as those who experienced conflict violence in pre-adulthood continue to raise children at the border who face ongoing insecurities—including legal status precarity and risks of statelessness.

## Supplementary Material

Supplement

## Figures and Tables

**FIGURE 1:**
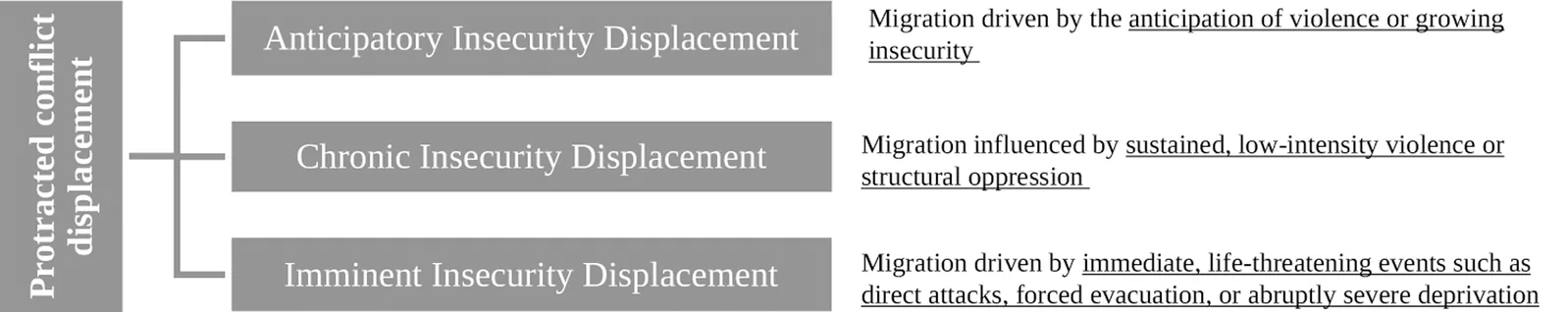
Conceptual framework outlining a typology of protracted conflict displacement based on the pressures, timings, and degrees of precarity under which displacement occurs

**FIGURE 2:**
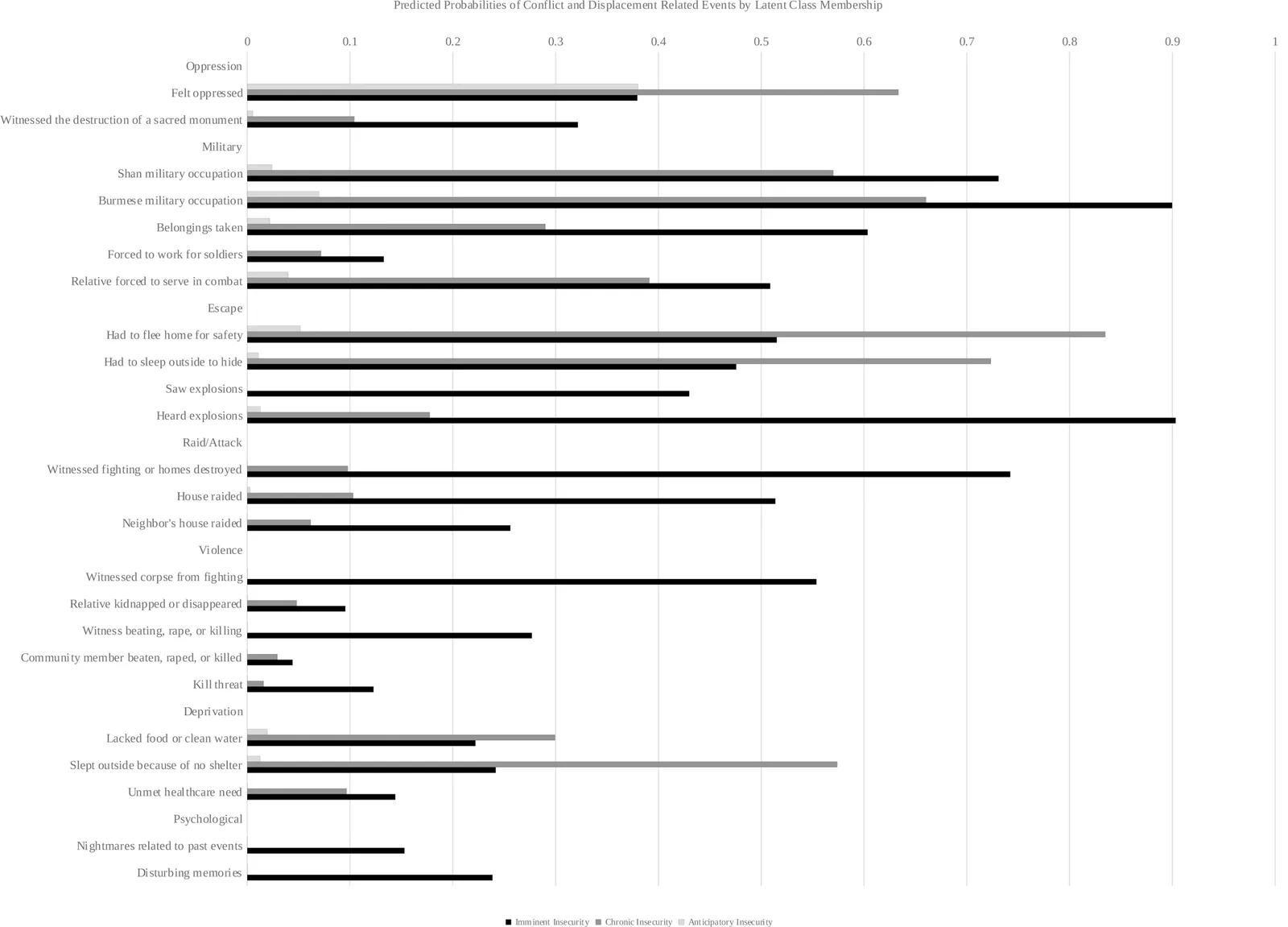
Latent class profiles of pre-displacement insecurities

**FIGURE 3:**
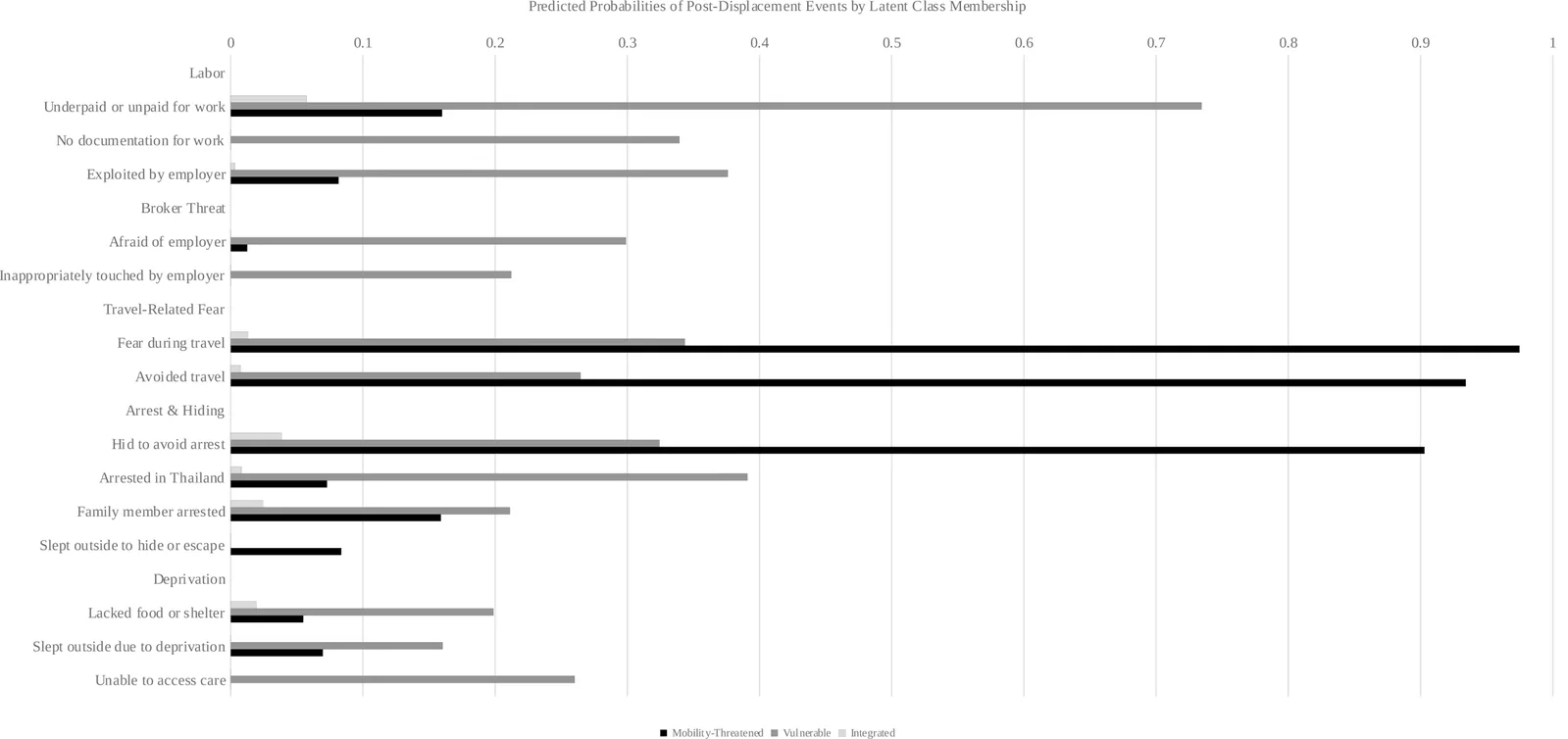
Latent class profiles of post-displacement insecurities

**FIGURE 4:**
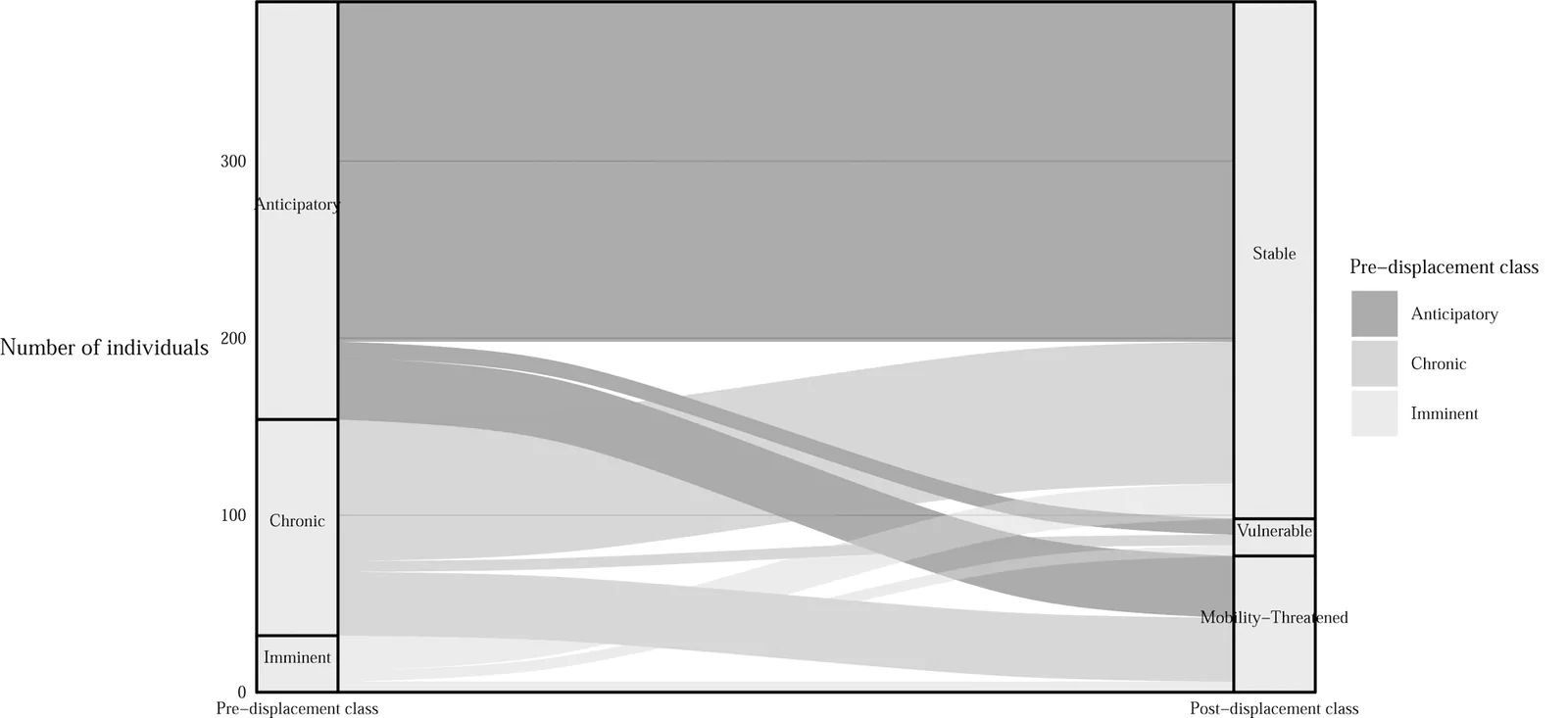
Migration trajectories showing continuity and divergence between pre- and post-displacement insecurities NOTE: Sankey diagram illustrating the flow of individuals across three latent classes of pre-displacement insecurity (Anticipatory, Chronic, and Imminent) and three classes of post-displacement insecurity (Stable/Integrated Migrants, Vulnerable Migrants, and Mobility-Threatened Migrants). While many Anticipatory Insecurity migrants transitioned into more stable circumstances post-migration, substantial proportions of Chronic and Imminent Insecurity migrants remained in or moved into insecure post-displacement contexts, reflecting continuity in precarity across borders. Corresponding flow counts, percentages, and statistical tests are showed in Supplement Table A5.

**FIGURE 5:**
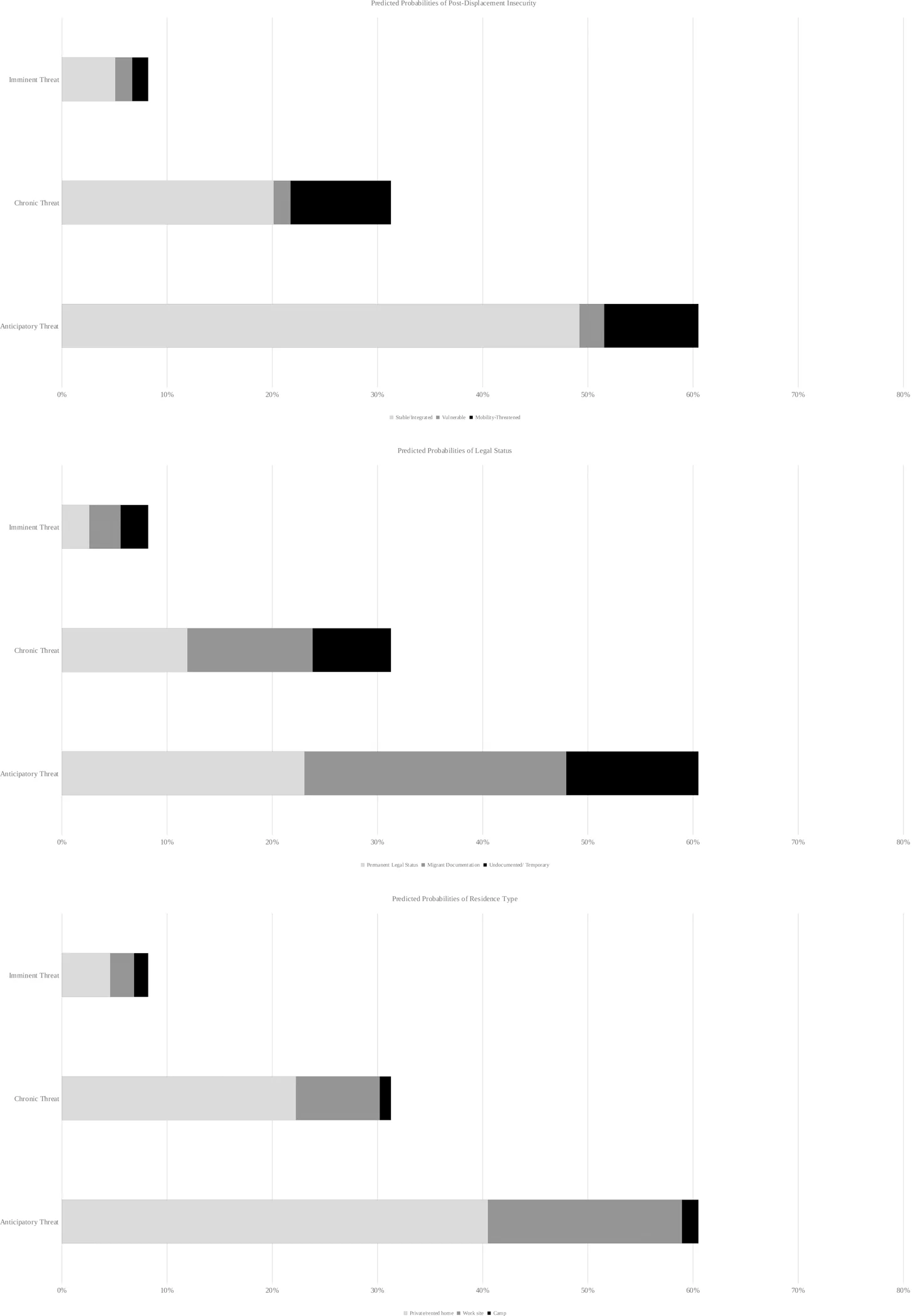
Post-displacement insecurity, legal status, and residence type by pre-displacement threat class NOTE: Stacked bar chart showing the distribution of predicted probabilities of post-displacement outcomes within each pre-displacement threat class, based on marginal standardization from multinomial regression models adjusting for educational attainment.

**TABLE 1:** Analytical sample characteristics (n=390).

	Mean	Standard Deviation

Maternal age	28.99	5.97
Age of migration from Myanmar to Thailand	16.52	7.84
Months since last childbirth	27.08	18.10

	Percent	

Ethnicity: Thai	4.62	
Ethnicity: Shan	75.13	
Ethnicity: Other highland ethnic minority	20.26	
Schooling completion: Less than primary	73.18	
Schooling completion: Primary	17.19	
Schooling completion: Secondary or higher	8.33	
Legal status: No legal documentation	23.59	
Legal status: Permanent legal status	37.44	
Legal status: Migrant documentation	38.97	
Residence: Private home	67.95	
Residence: Work site	28.21	
Residence: Camp	3.85	
Income source: Agriculture	17.05	
Income source: Construction	8.53	
Income source: Service	0.52	
Income source: Procurement transportation communication	15.76	
Income source: Agricultural wage labor	35.66	
Income source: Factory work	20.41	
Income source: Unreported	2.07	

**TABLE 2:** Adjusted Relative Risk Ratios from Multinomial Logistic Regression Models Predicting Post-Displacement Outcomes by Pre-Displacement Insecurity Class and Education Level.

	Post-Displacement: Vulnerable Migrants	Post- Displacement: Mobility-Threatened	Legal Status: Migrant Documentation	Legal Status: Undocumented/ Temporary	Residence: Worksite	Residence: Camp

**Pre-displacement**: Anticipatory Insecurity	(REF)	(REF)	(REF)	(REF)	(REF)	(REF)
**Pre-displacement**: Chronic Insecurity	1.66 (0.57, 4.85)	2.61** (1.52, 4.48)	0.91 (0.53, 1.58)	1.14 (0.63, 2.08)	0.77 (0.46, 1.30)	1.25 (0.34, 4.56)
**Pre-displacement**: Imminent Insecurity	6.70** (2.11, 21.24)	1.64 (0.62, 4.37)	1.05 (0.39, 2.83)	1.85 (0.68, 5.01)	1.05 (0.42, 2.63)	7.76** (2.26, 26.63)
**Education**: Less than Primary	(REF)	(REF)	(REF)	(REF)	(REF)	(REF)
**Education**: Primary	1.80 (0.60, 5.43)	0.83 (0.40, 1.70)	0.16*** (0.08, 0.35)	0.69 (0.36, 1.32)	0.45* (0.23, 0.91)	2.23 (0.60, 8.31)
**Education**: Secondary+	0.95 (0.21, 4.34)	0.70 (0.28, 1.76)	0.09*** (0.03, 0.27)	0.35* (0.15, 0.85)	0.06** (0.01, 0.41)	1.95 (0.56, 6.81)

Wald test of coefficients indicated by:

* *p* < *0.05*

** *p* < *0.01*

*** *p* < *0.001*

## Data Availability

The data that support the findings of this study are available on request from the corresponding author, Stephanie Koning. The data are not publicly available due to them containing information that could compromise research participant privacy.
