# Supplementary material for: Anticipatory, Chronic, and Imminent: A Typology of Insecurities Underlying Protracted Conflict Displacement and Its Implications
Source: Popul Dev Rev. Author manuscript; Available in PMC 2026 Nov 7. (PMC13742193; doi:10.1111/padr.70049)
Supplement: Supplementary file 1 [file NIHMS2173808-supplement-Supplement.pdf]

Appendix. Supplemental Materials.

Latent, Chronic, and Imminent: A Typology of Threats Underlying Protracted Conflict Displacement

Table of Contents

Online Supplemental Material. .... *Error! Bookmark not defined.*

Table A1. Analytical sample characteristics and item response (n=390). ***2***

Table A2. Summary of marginal predicted probabilities of pre-displacement threats by latent class. ***3***

Table A3. Summary of Pre-Displacement Threat Indicator Marginal Predicted Probabilities by Latent Class. ***5***

Table A4. Cross tabulation of pre- and post-displacement threat classes. ***7***

Table A5. Adjusted Predicted Probabilities of Post-Displacement Outcomes by Pre-Displacement Threat Typology. ***8***

Table A1. Analytical sample characteristics and item response (n=390).

	Mean	Standard Deviation	Median	IQR	Minimum	Maximum	Count
Maternal age	28.992	5.968	29	10	18	40	390
Age of migration from Myanmar to Thailand	16.520	7.843	17	8	-1	36	300
Months since last childbirth	27.085	18.096	24	27	0	84	365
	**Percent**						
Ethnicity: Thai	4.6		0	0	0	1	390
Ethnicity: Shan	75.1		1	0	0	1	390
Ethnicity: Other highland ethnic minority	20.3		0	0	0	1	390
Schooling completion: Less than primary	73.2		1	1	0	1	384
Schooling completion: Primary	17.2		0	0	0	1	384
Schooling completion: Secondary or higher	8.3		0	0	0	1	384
Legal status: No legal documentation	23.6		0	0	0	1	390
Legal status: Permanent legal status	37.4		0	1	0	1	390
Legal status: Migrant documentation	39		0	1	0	1	390
Residence: Private home	67.9		1	1	0	1	390
Residence: Work site	28.2		0	1	0	1	390
Residence: Camp	3.8		0	0	0	1	390
Income source: None	0.3		0	0	0	1	387
Income source: Agriculture	17.2		0	0	0	1	383
Income source: Construction	8.6		0	0	0	1	384
Income source: Service	0.5		0	0	0	1	388
Income source: Procurement transportation communication	16		0	0	0	1	382
Income source: Agricultural wage labor	36.1		0	1	0	1	382
Income source: Factory work	20.6		0	0	0	1	383
Income source: Government or education	1.6		0	0	0	1	249

Table A2. **Summary of marginal predicted probabilities of pre-displacement threats by latent class.**

		**Anticipatory**				**Chronic**			
				**95% CI**				**95% CI**	
**Domain**	**Variable**	**Mean**	**SE**	**Lower**	**Upper**	**Mean**	**SE**	**Lower**	**Upper**
**Oppression**	Felt oppressed	0.358	0.0328	0.297	0.425	0.55	0.0475	0.457	0.641
	Witnessed the destruction of a sacred monument	0.006	0.0058	0.001	0.04	0.101	0.0318	0.054	0.183
**Military**	Shan military occupation	0.03	0.0205	0.008	0.109	0.582	0.0532	0.475	0.681
	Burmese military occupation	0.083	0.0253	0.045	0.148	0.659	0.0501	0.555	0.749
	Relatived forced to work for soldiers	0.018	0.0096	0.007	0.05	0.313	0.0514	0.222	0.421
	Relative forced to serve in combat	0.034	0.014	0.015	0.075	0.399	0.0482	0.309	0.496
**Escape**	Had to flee home for safety	0.048	0.0168	0.024	0.094	0.645	0.0545	0.532	0.743
	Had to sleep outside to hide	0.01	0.0073	0.002	0.042	0.497	0.0557	0.39	0.605
	Heard explosions	0.014	0.0086	0.004	0.046	0.192	0.046	0.118	0.299
**Raid/Attack**	Witnessed fighing or homes destroyed	1.00E-05	0.13	0.037	0.073	0.221	0.859	0.0953	0.566
	House raided	0.004	0.0043	0.0005	0.032	0.115	0.0313	0.067	0.192
	Neighbor's house raided	1.00E-05				0.055	0.0226	0.024	0.12
**Violence**	Witnessed corpse from fighting	1.00E-05				6.00E-07	1.00E-07	4.00E-07	8.00E-07
	Relative kidnapped or disappeared	1.00E-05				0.051	0.0216	0.022	0.114
	Community member beaten, raped, or killed	1.00E-05				1.00E-05			
**Deprivation**	Lacked food or shelter	0.02	0.01	0.007	0.053	0.26	0.0503	0.174	0.37
	Slept outside because of no shelter	0.006	0.0084	0.0004	0.082	0.551	0.0595	0.434	0.663
**Psychological**	Nightmares related to past events	1.00E-05				6.00E-07	5.80E-05	4.50E-05	6.80E-05
	Disturbing memories	1.00E-05				6.00E-07	6.40E-05	4.50E-05	7.00E-05

		Imminent			
				95% CI	
**Domain**	**Variable**	**Mean**	**SE**	**Lower**	**Upper**
**Oppression**	Felt oppressed	0.533	0.0969	0.348	0.71
	Witnessed the destruction of a sacred monument	0.446	0.0983	0.27	0.637
**Military**	Shan military occupation	0.717	0.0854	0.526	0.853
	Burmese military occupation	0.858	0.0686	0.667	0.948
	Relatived forced to work for soldiers	0.636	0.0946	0.439	0.795
	Relative forced to serve in combat	0.621	0.1007	0.415	0.791
**Escape**	Had to flee home for safety	0.577	0.1083	0.364	0.765
	Had to sleep outside to hide	0.528	0.116	0.31	0.736
	Heard explosions	0.819	0.0741	0.63	0.924
**Raid/Attack**	Witnessed fighing or homes destroyed	0.966			
	House raided	0.645	0.1174	0.4	0.833
	Neighbor's house raided	0.524	0.1056	0.325	0.716
**Violence**	Witnessed corpse from fighting	0.766	0.1386	0.418	0.937
	Relative kidnapped or disappeared	0.229	0.0819	0.107	0.424
	Community member beaten, raped, or killed	0.364	0.125	0.166	0.623
**Deprivation**	Lacked food or shelter	0.349	0.1211	0.159	0.604
	Slept outside because of no shelter	0.274	0.1221	0.102	0.557
**Psychological**	Nightmares related to past events	0.18	0.0859	0.066	0.407
	Disturbing memories	0.289	0.1302	0.105	0.585

Table A3. **Summary of Pre-Displacement Threat Indicator Marginal Predicted Probabilities by Latent Class.**

Domain	Indicator	Integrated	SE	95% CI	Vulnerable	SE	95% CI	Mobility-Threatened	SE	95% CI
**Labor**	Underpaid or unpaid for work	0.057	0.015	[0.03, 0.09]	0.734	0.133	[0.42, 0.91]	0.16	0.061	[0.07, 0.32]
	No documentation for work	4.04E-09			0.339	0.132	[0.14, 0.62]	1.05E-08		
	Exploited by broker	0.003	0.004	[0.00, 0.03]	0.376	0.165	[0.13, 0.70]	0.082	0.049	[0.02, 0.24]
**Broker Threat**	Afraid of broker	5.60E-09			0.299	0.133	[0.11, 0.60]	0.013	0.013	[0.00, 0.09]
	Inappropriately touched by broker	1.52E-08			0.212	0.115	[0.07, 0.51]	2.46E-08	3.14E-09	[1.91e-08,3.16e-08]
**Travel-Related Fear**	Fear during travel	0.013	0.007	[0.00, 0.04]	0.344	0.179	[0.10, 0.71]	0.975	0.027	[0.82, 1.00]
	Avoided authorities	0.007	0.006	[0.00, 0.03]	0.265	0.156	[0.07, 0.64]	0.934	0.033	[0.83, 0.98]
**Arrest & Hiding**	Hid to avoid arrest	0.038	0.012	[0.02, 0.07]	0.324	0.165	[0.10, 0.68]	0.903	0.037	[0.80, 0.96]
	Arrested in Thailand	0.008	0.009	[0.00, 0.07]	0.391	0.138	[0.17, 0.67]	0.073	0.043	[0.02, 0.22]
	Family member arrested	0.024	0.01	[0.01, 0.06]	0.211	0.165	[0.04, 0.65]	0.159	0.06	[0.07, 0.31]
	Slept outside to hide or escape	1.08E-08			2.22E-09			0.084	0.032	[0.04, 0.17]
**Deprivation**	Lacked food or shelter	0.019	0.01	[0.01, 0.05]	0.199	0.272	[0.01, 0.88]	0.055	0.04	[0.01, 0.21]
	Slept outside due to deprivation	2.81E-09			0.16	0.228	[0.01, 0.84]	0.07	0.04	[0.02, 0.20]

**Access to Care**	Unable to access care	5.23E-09	0.26	0.132	[0.08, 0.57]	2.66E-08	3.37E-09	[2.07e-08, 3.41e-08]

Table A4. **Cross tabulation of pre- and post-displacement threat classes.**

**Pre-Displacement Class**	**Stable/Integrated**	**Vulnerable**	**Mobility-Threatened**	**Total**
**Anticipatory Threat**	191 (81.6%)	9 (3.9%)	34 (14.5%)	234
**Chronic Threat**	85 (66.4%)	7 (5.5%)	36 (28.1%)	128
**Imminent Threat**	16 (57.1%)	5 (17.9%)	7 (25.0%)	28
**Total**	292 (74.9%)	21 (5.4%)	77 (19.7%)	390

Pearson $\chi^2(4) = 21.11$, $p < 0.001$

Table A5. **Adjusted Predicted Probabilities of Post-Displacement Outcomes by Pre-Displacement Threat Typology.**

	Anticipatory Threat			Chronic Threat			Imminent Threat		
Outcome	Point estimate	Lower 95% CI	Upper 95% CI	Point estimate	Lower 95% CI	Upper 95% CI	Point estimate	Lower 95% CI	Upper 95% CI
Stable/Integrated Migrants	0.816	0.766	0.866	0.653	0.569	0.738	0.566	0.382	0.750
Vulnerable Migrants	0.039	0.014	0.064	0.057	0.016	0.098	0.185	0.040	0.329
Mobility-Threatened Migrants	0.145	0.099	0.191	0.290	0.209	0.370	0.249	0.091	0.407
Permanent Legal Status	0.371	0.314	0.428	0.408	0.321	0.494	0.281	0.110	0.452
Migrant Documentation	0.420	0.360	0.480	0.374	0.291	0.457	0.318	0.158	0.478
Undocumented/Temporary Status	0.209	0.156	0.262	0.219	0.146	0.291	0.401	0.222	0.579
Residence: Private Home	0.671	0.612	0.729	0.695	0.615	0.776	0.603	0.419	0.786
Residence: Work Site	0.303	0.246	0.361	0.272	0.193	0.350	0.217	0.063	0.370
Residence: Camp	0.026	0.006	0.046	0.033	0.001	0.065	0.181	0.049	0.313

Note: Values represent marginal predicted probabilities from three multinomial logistic regression models with robust standard errors (Stata 18), adjusted for education level. Each column represents a pre-displacement threat typology, and rows denote predicted probabilities of each post-displacement outcome within that column. 95% confidence intervals are reported in separate columns.
